# Supplementary material for: Successional Shifts in Soil C:N:P stoichiometry and its environmental controls during forest restoration in Huize County, Qujing, Yunnan Province
Source: PLoS One. 2026 Aug 21;21(8):e0356261. doi: 10.1371/journal.pone.0356261 (PMC13496410; doi:10.1371/journal.pone.0356261)
Supplement: Supplementary file 2 [file pone.0356261.s004.zip › Data and Code/RDA/mixed conifer–broadleaf forest/RDA-2.pdf]

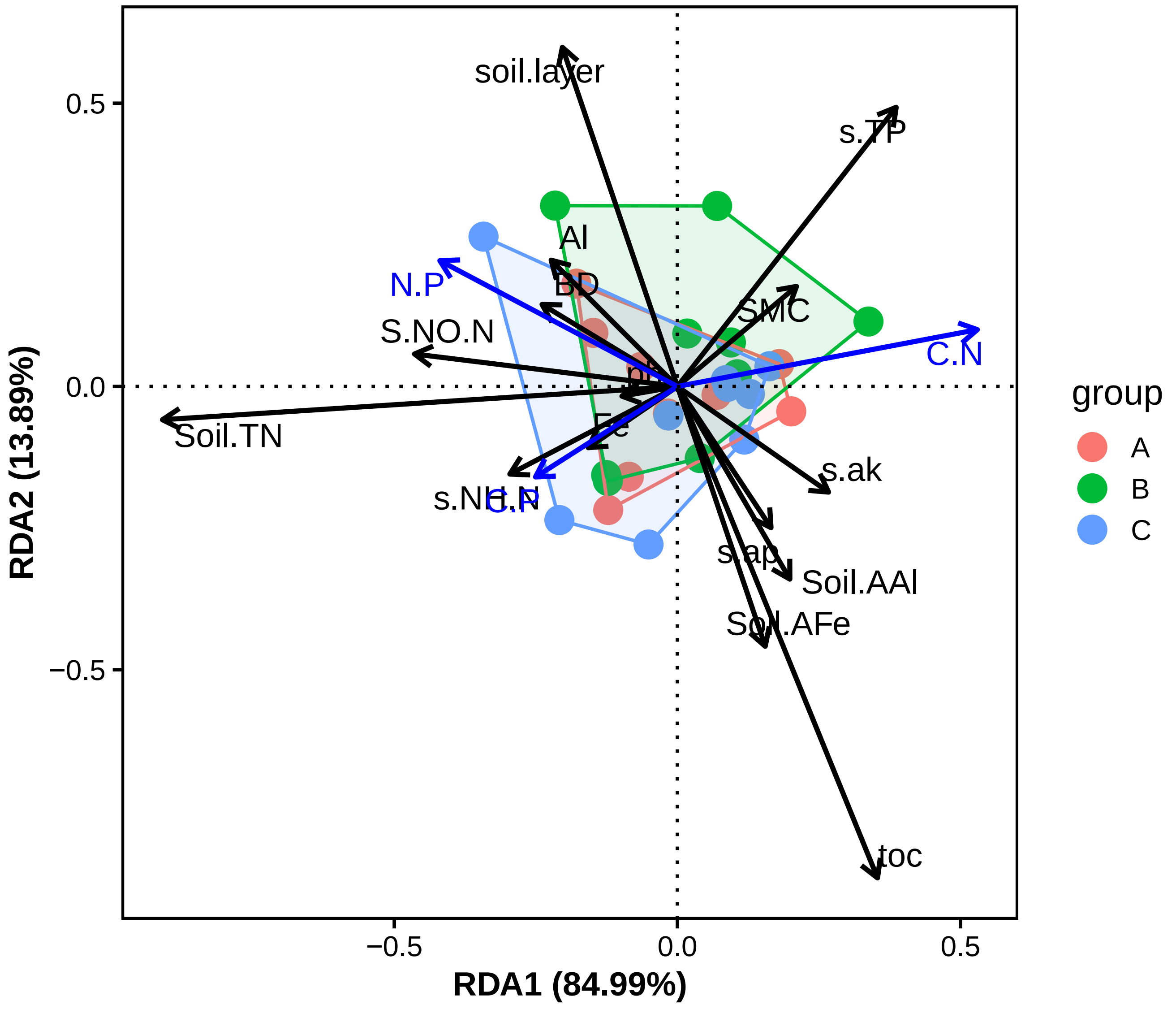

soil.layer
s.TP
Al
BD
N.P
S.NO.N
SMC
C.N
pH
Soil.TN
Fe
s.ak
s.NH.N
C.P
s.ap
Soil.AAl
Soil.AFe
toc
0.5
0.0
−0.5
−0.5
0.0
0.5
RDA2 (13.89%)
RDA1 (84.99%)
group
A
B
C
